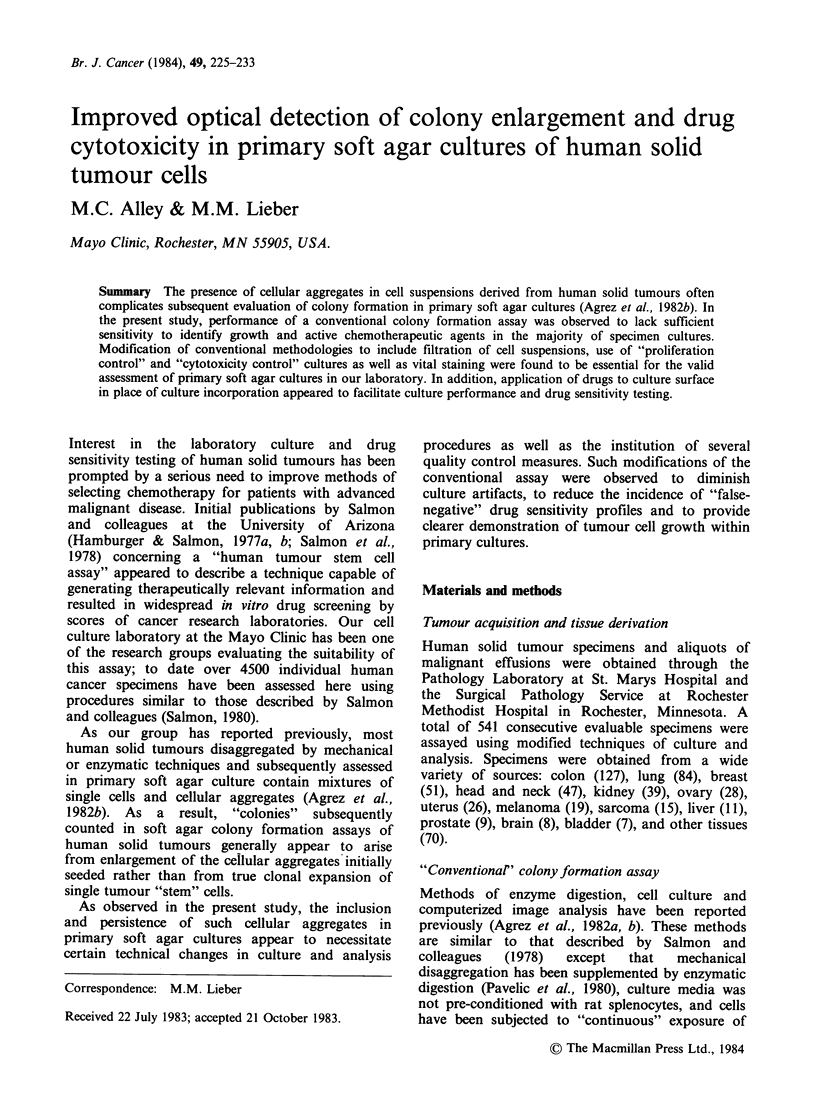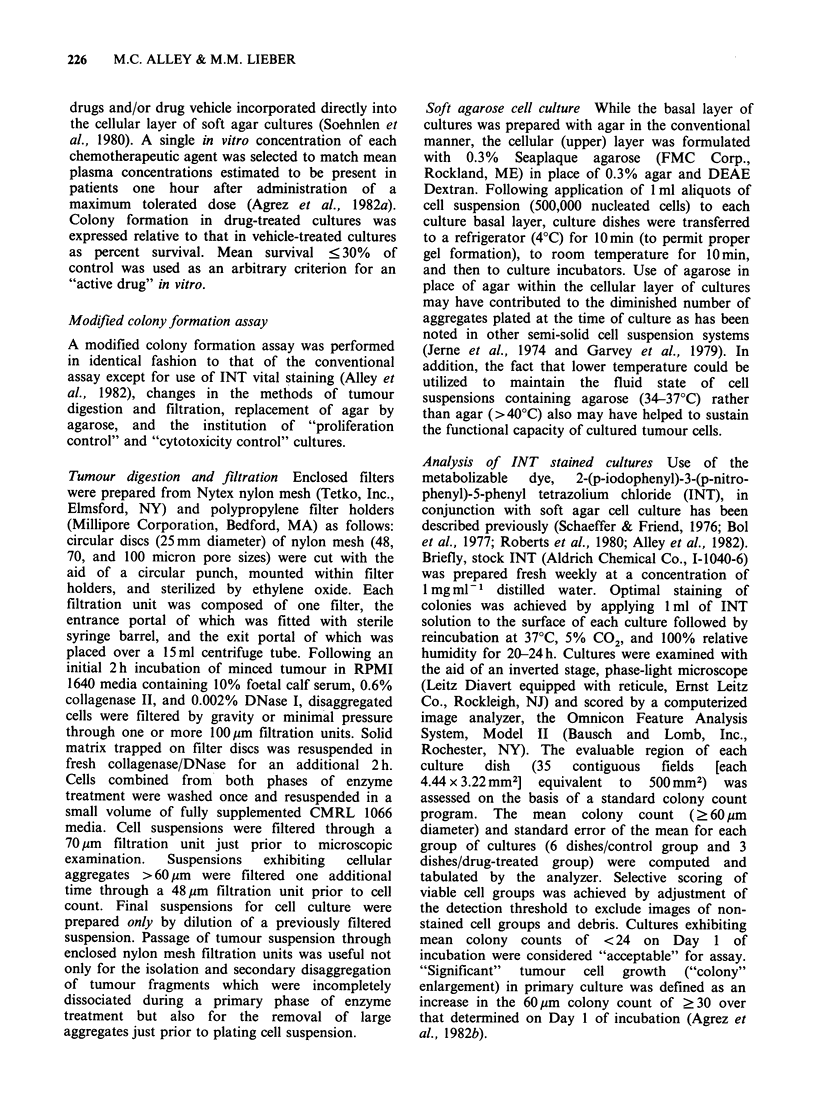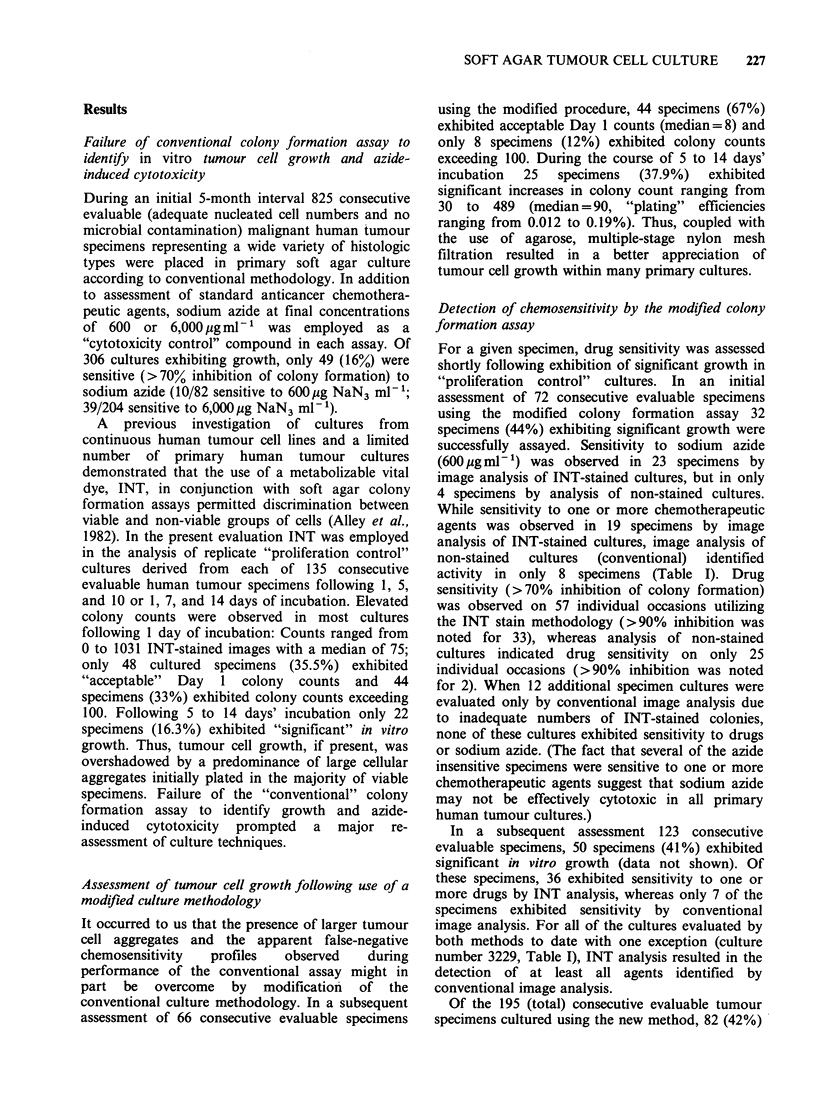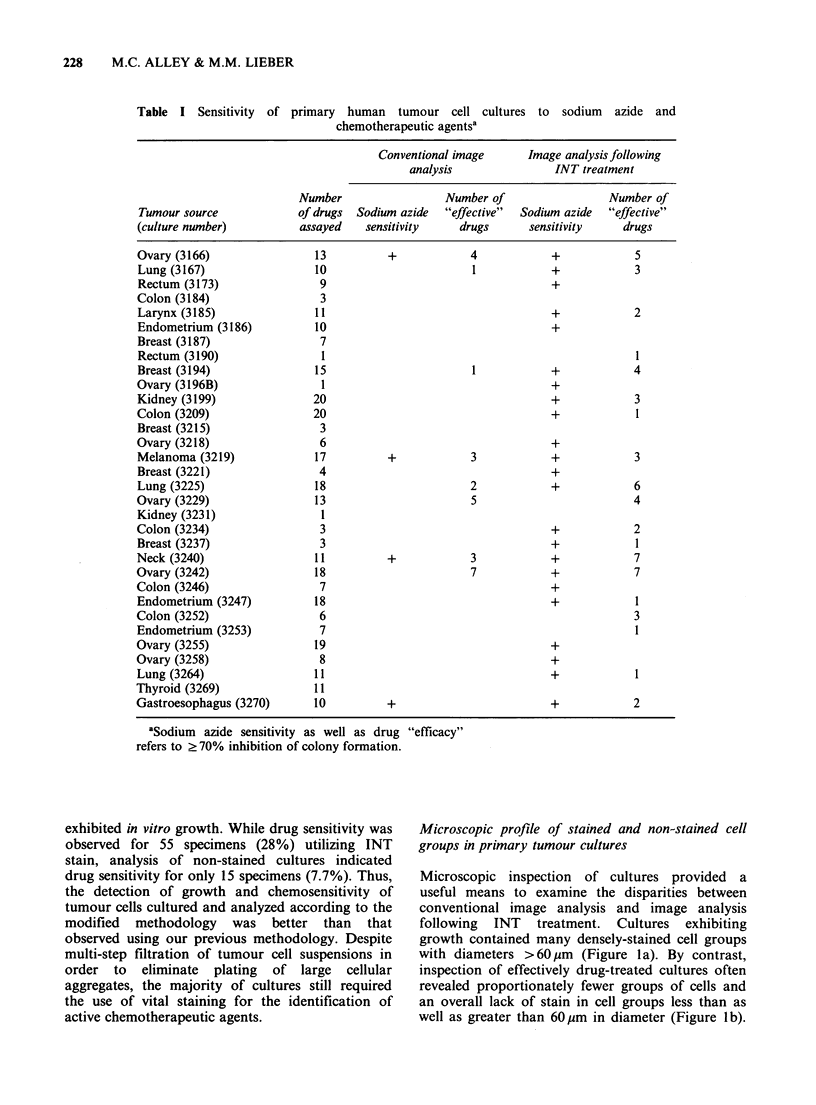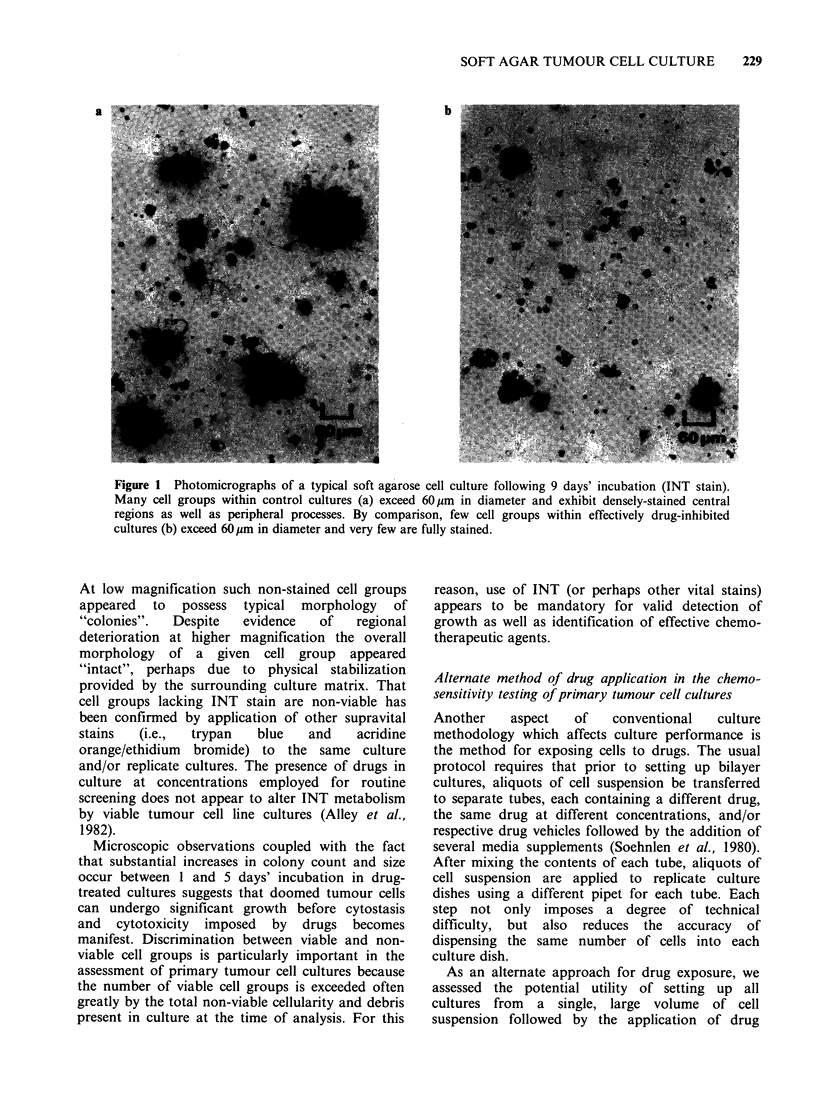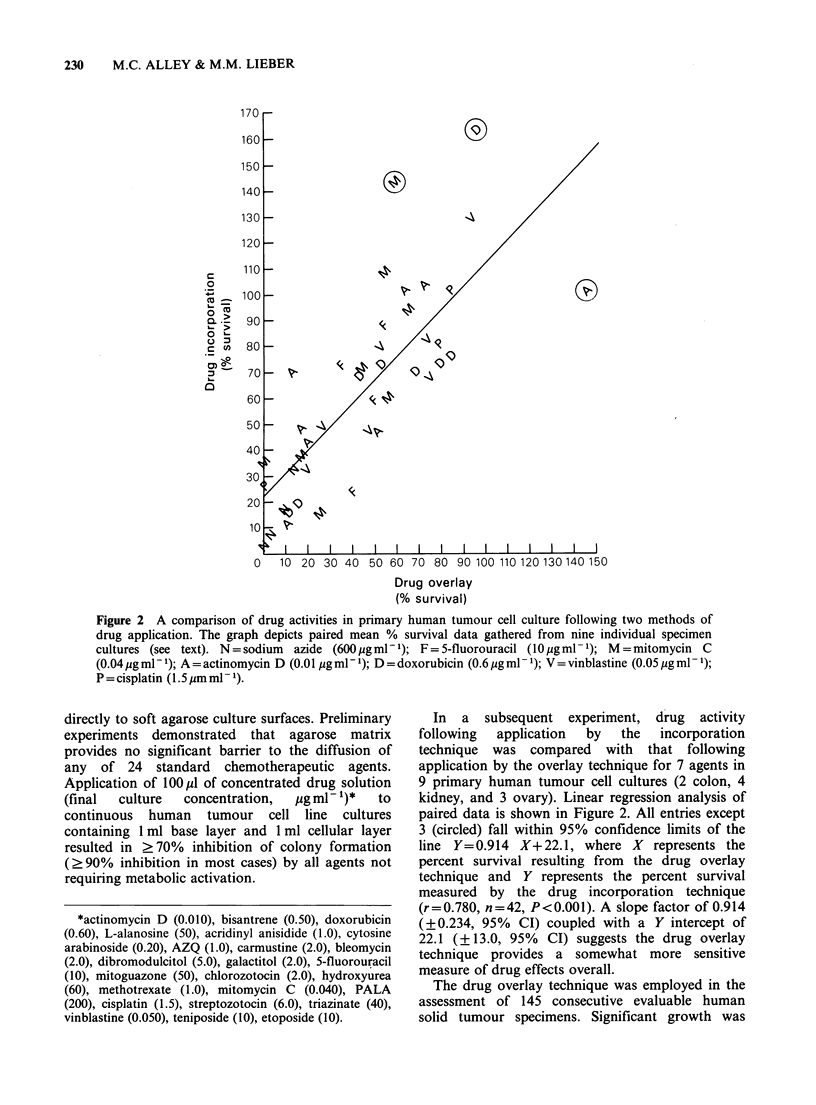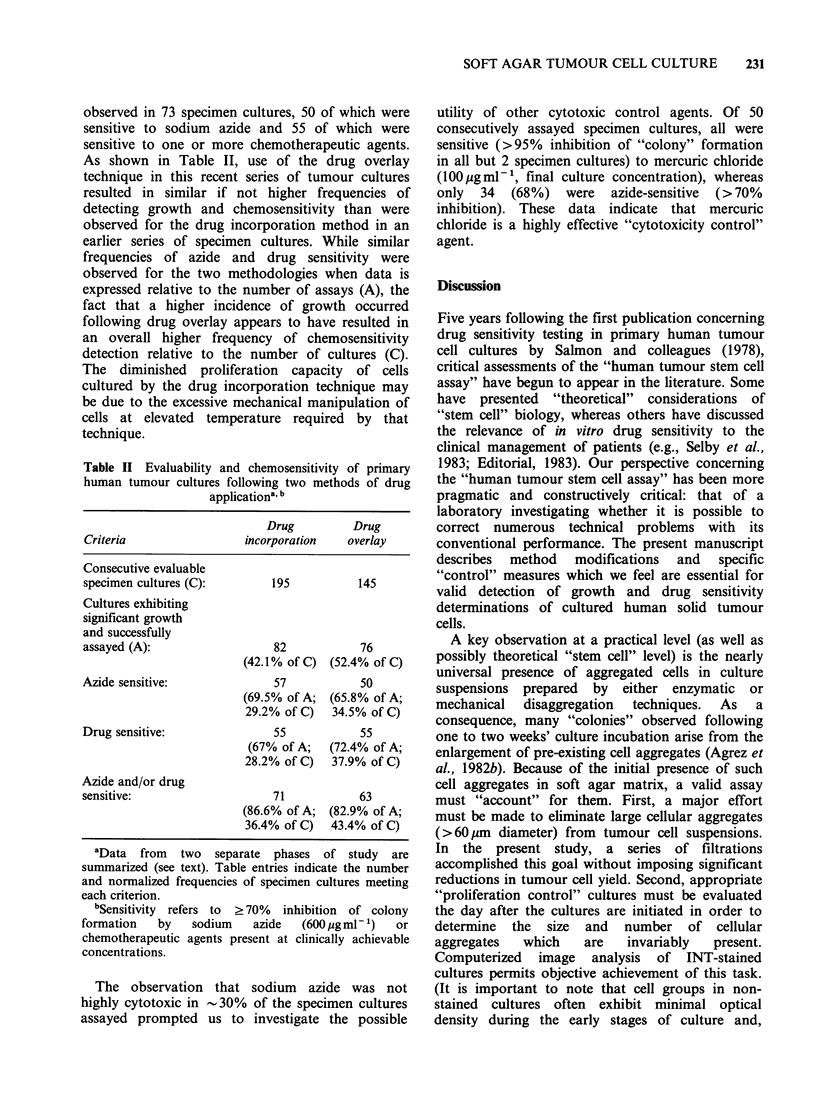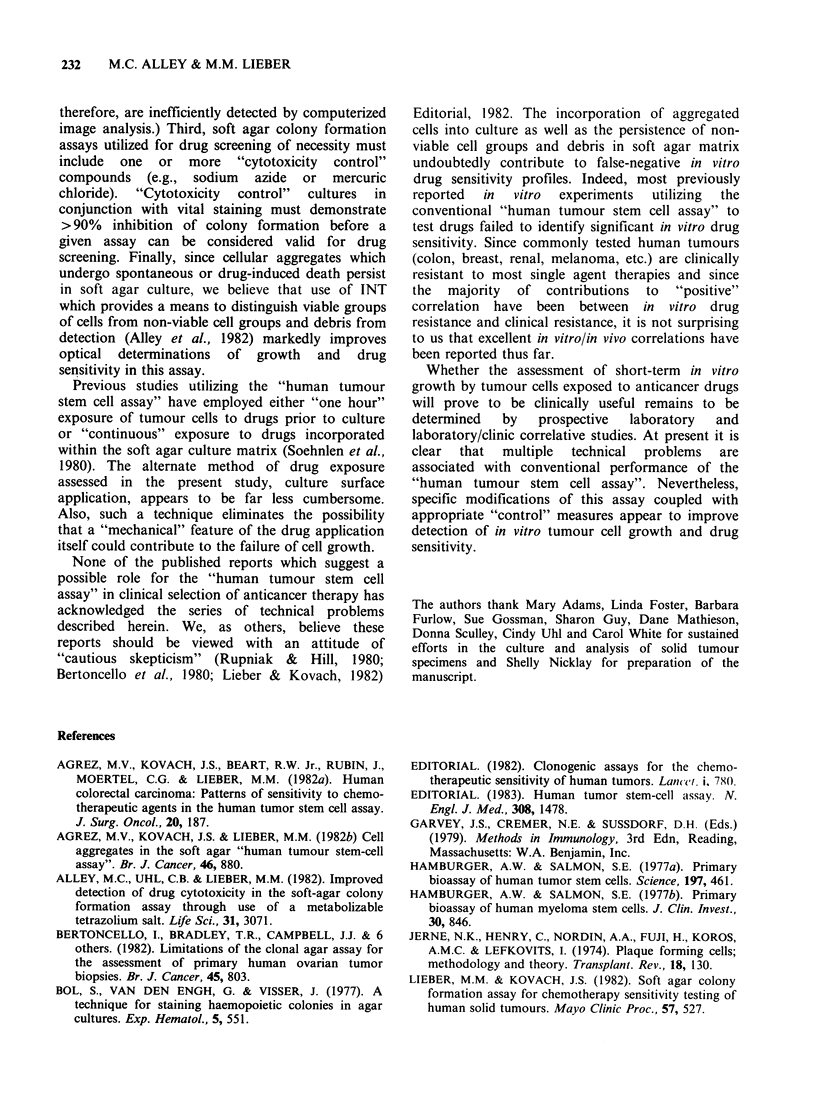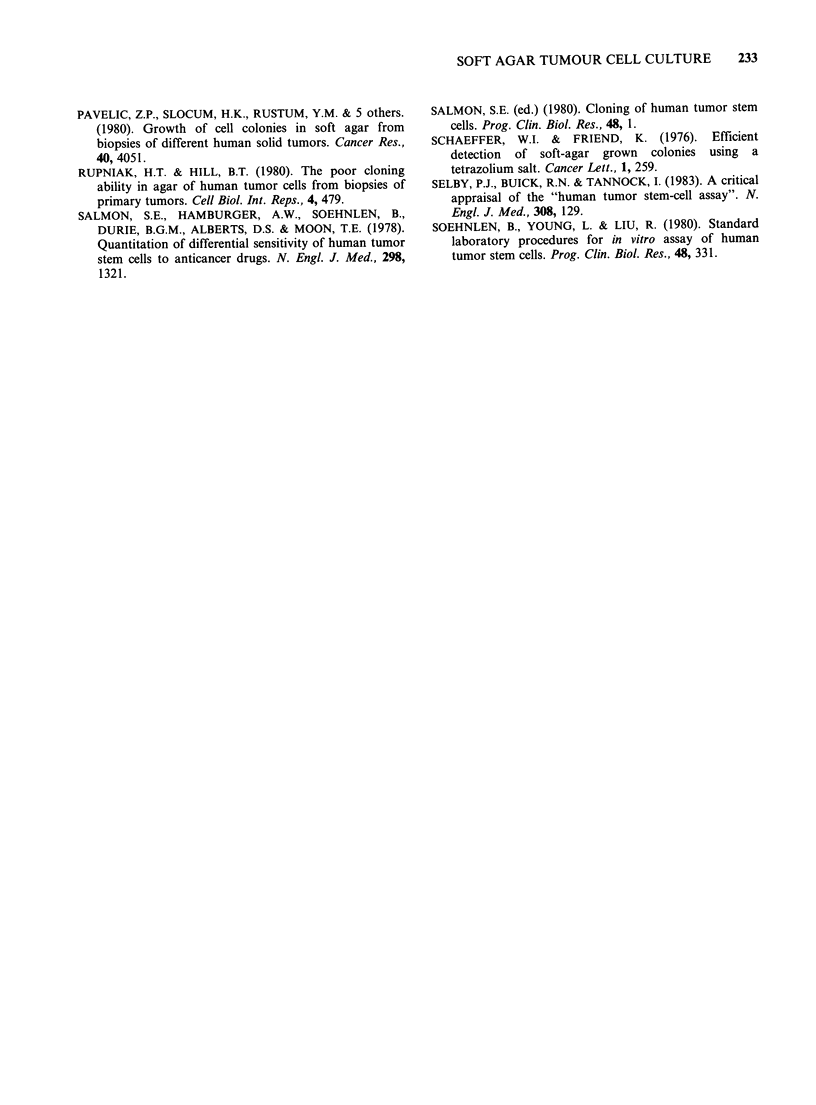# Improved optical detection of colony enlargement and drug cytotoxicity in primary soft agar cultures of human solid tumour cells.

**DOI:** 10.1038/bjc.1984.35

**Published:** 1984-02

**Authors:** M. C. Alley, M. M. Lieber

## Abstract

**Images:**


*Br. J. Cancer* (1984), **49**, 225–233

### Improved optical detection of colony enlargement and drug cytotoxicity in primary soft agar cultures of human solid tumour cells

M.C. Alley & M.M. Lieber

*Mayo Clinic, Rochester, MN 55905, USA.*

**Summary** The presence of cellular aggregates in cell suspensions derived from human solid tumours often complicates subsequent evaluation of colony formation in primary soft agar cultures (Agrez *et al.*, 1982*b*). In the present study, performance of a conventional colony formation assay was observed to lack sufficient sensitivity to identify growth and active chemotherapeutic agents in the majority of specimen cultures. Modification of conventional methodologies to include filtration of cell suspensions, use of "proliferation control" and "cytotoxicity control" cultures as well as vital staining were found to be essential for the valid assessment of primary soft agar cultures in our laboratory. In addition, application of drugs to culture surface in place of culture incorporation appeared to facilitate culture performance and drug sensitivity testing.

Interest in the laboratory culture and drug sensitivity testing of human solid tumours has been prompted by a serious need to improve methods of selecting chemotherapy for patients with advanced malignant disease. Initial publications by Salmon and colleagues at the University of Arizona (Hamburger & Salmon, 1977*a*, *b*; Salmon *et al.*, 1978) concerning a "human tumour stem cell assay" appeared to describe a technique capable of generating therapeutically relevant information and resulted in widespread *in vitro* drug screening by scores of cancer research laboratories. Our cell culture laboratory at the Mayo Clinic has been one of the research groups evaluating the suitability of this assay; to date over 4500 individual human cancer specimens have been assessed here using procedures similar to those described by Salmon and colleagues (Salmon, 1980).

As our group has reported previously, most human solid tumours disaggregated by mechanical or enzymatic techniques and subsequently assessed in primary soft agar culture contain mixtures of single cells and cellular aggregates (Agrez *et al.*, 1982*b*). As a result, "colonies" subsequently counted in soft agar colony formation assays of human solid tumours generally appear to arise from enlargement of the cellular aggregates initially seeded rather than from true clonal expansion of single tumour "stem" cells.

As observed in the present study, the inclusion and persistence of such cellular aggregates in primary soft agar cultures appear to necessitate certain technical changes in culture and analysis procedures as well as the institution of several quality control measures. Such modifications of the conventional assay were observed to diminish culture artifacts, to reduce the incidence of "false-negative" drug sensitivity profiles and to provide clearer demonstration of tumour cell growth within primary cultures.

Correspondence: M.M. Lieber

Received 22 July 1983; accepted 21 October 1983.

### Materials and methods

### *Tumour acquisition and tissue derivation*

Human solid tumour specimens and aliquots of malignant effusions were obtained through the Pathology Laboratory at St. Marys Hospital and the Surgical Pathology Service at Rochester Methodist Hospital in Rochester, Minnesota. A total of 541 consecutive evaluable specimens were assayed using modified techniques of culture and analysis. Specimens were obtained from a wide variety of sources: colon (127), lung (84), breast (51), head and neck (47), kidney (39), ovary (28), uterus (26), melanoma (19), sarcoma (15), liver (11), prostate (9), brain (8), bladder (7), and other tissues (70).

### *"Conventional" colony formation assay*

Methods of enzyme digestion, cell culture and computerized image analysis have been reported previously (Agrez *et al.*, 1982*a*, *b*). These methods are similar to that described by Salmon and colleagues (1978) except that mechanical disaggregation has been supplemented by enzymatic digestion (Pavelic *et al.*, 1980), culture media was not pre-conditioned with rat splenocytes, and cells have been subjected to "continuous" exposure of

© The Macmillan Press Ltd., 1984

drugs and/or drug vehicle incorporated directly into the cellular layer of soft agar cultures (Soehnlen *et al.*, 1980). A single *in vitro* concentration of each chemotherapeutic agent was selected to match mean plasma concentrations estimated to be present in patients one hour after administration of a maximum tolerated dose (Agrez *et al.*, 1982*a*). Colony formation in drug-treated cultures was expressed relative to that in vehicle-treated cultures as percent survival. Mean survival $\leq 30\%$ of control was used as an arbitrary criterion for an "active drug" *in vitro*.

### *Modified colony formation assay*

A modified colony formation assay was performed in identical fashion to that of the conventional assay except for use of INT vital staining (Alley *et al.*, 1982), changes in the methods of tumour digestion and filtration, replacement of agar by agarose, and the institution of "proliferation control" and "cytotoxicity control" cultures.

*Tumour digestion and filtration* Enclosed filters were prepared from Nytex nylon mesh (Tetko, Inc., Elmsford, NY) and polypropylene filter holders (Millipore Corporation, Bedford, MA) as follows: circular discs (25 mm diameter) of nylon mesh (48, 70, and 100 micron pore sizes) were cut with the aid of a circular punch, mounted within filter holders, and sterilized by ethylene oxide. Each filtration unit was composed of one filter, the entrance portal of which was fitted with sterile syringe barrel, and the exit portal of which was placed over a 15 ml centrifuge tube. Following an initial 2 h incubation of minced tumour in RPMI 1640 media containing 10% foetal calf serum, 0.6% collagenase II, and 0.002% DNase I, disaggregated cells were filtered by gravity or minimal pressure through one or more 100 $\mu$m filtration units. Solid matrix trapped on filter discs was resuspended in fresh collagenase/DNase for an additional 2 h. Cells combined from both phases of enzyme treatment were washed once and resuspended in a small volume of fully supplemented CMRL 1066 media. Cell suspensions were filtered through a 70 $\mu$m filtration unit just prior to microscopic examination. Suspensions exhibiting cellular aggregates $>60\,\mu$m were filtered one additional time through a 48 $\mu$m filtration unit prior to cell count. Final suspensions for cell culture were prepared *only* by dilution of a previously filtered suspension. Passage of tumour suspension through enclosed nylon mesh filtration units was useful not only for the isolation and secondary disaggregation of tumour fragments which were incompletely dissociated during a primary phase of enzyme treatment but also for the removal of large aggregates just prior to plating cell suspension.

*Soft agarose cell culture* While the basal layer of cultures was prepared with agar in the conventional manner, the cellular (upper) layer was formulated with 0.3% Seaplaque agarose (FMC Corp., Rockland, ME) in place of 0.3% agar and DEAE Dextran. Following application of 1 ml aliquots of cell suspension (500,000 nucleated cells) to each culture basal layer, culture dishes were transferred to a refrigerator (4°C) for 10 min (to permit proper gel formation), to room temperature for 10 min, and then to culture incubators. Use of agarose in place of agar within the cellular layer of cultures may have contributed to the diminished number of aggregates plated at the time of culture as has been noted in other semi-solid cell suspension systems (Jerne *et al.*, 1974 and Garvey *et al.*, 1979). In addition, the fact that lower temperature could be utilized to maintain the fluid state of cell suspensions containing agarose (34–37°C) rather than agar ($>40$°C) also may have helped to sustain the functional capacity of cultured tumour cells.

*Analysis of INT stained cultures* Use of the metabolizable dye, 2-(p-iodophenyl)-3-(p-nitrophenyl)-5-phenyl tetrazolium chloride (INT), in conjunction with soft agar cell culture has been described previously (Schaeffer & Friend, 1976; Bol *et al.*, 1977; Roberts *et al.*, 1980; Alley *et al.*, 1982). Briefly, stock INT (Aldrich Chemical Co., I-1040-6) was prepared fresh weekly at a concentration of 1 mg ml$^{-1}$ distilled water. Optimal staining of colonies was achieved by applying 1 ml of INT solution to the surface of each culture followed by reincubation at 37°C, 5% $CO_2$, and 100% relative humidity for 20–24 h. Cultures were examined with the aid of an inverted stage, phase-light microscope (Leitz Diavert equipped with reticule, Ernst Leitz Co., Rockleigh, NJ) and scored by a computerized image analyzer, the Omnicon Feature Analysis System, Model II (Bausch and Lomb, Inc., Rochester, NY). The evaluable region of each culture dish (35 contiguous fields [each $4.44 \times 3.22$ mm$^2$] equivalent to 500 mm$^2$) was assessed on the basis of a standard colony count program. The mean colony count ($\geq 60\,\mu$m diameter) and standard error of the mean for each group of cultures (6 dishes/control group and 3 dishes/drug-treated group) were computed and tabulated by the analyzer. Selective scoring of viable cell groups was achieved by adjustment of the detection threshold to exclude images of non-stained cell groups and debris. Cultures exhibiting mean colony counts of $<24$ on Day 1 of incubation were considered "acceptable" for assay. "Significant" tumour cell growth ("colony" enlargement) in primary culture was defined as an increase in the 60 $\mu$m colony count of $\geq 30$ over that determined on Day 1 of incubation (Agrez *et al.*, 1982*b*).

### Results

*Failure of conventional colony formation assay to identify* in vitro *tumour cell growth and azide-induced cytotoxicity*

During an initial 5-month interval 825 consecutive evaluable (adequate nucleated cell numbers and no microbial contamination) malignant human tumour specimens representing a wide variety of histologic types were placed in primary soft agar culture according to conventional methodology. In addition to assessment of standard anticancer chemotherapeutic agents, sodium azide at final concentrations of 600 or 6,000 $\mu g\ ml^{-1}$ was employed as a "cytotoxicity control" compound in each assay. Of 306 cultures exhibiting growth, only 49 (16%) were sensitive (>70% inhibition of colony formation) to sodium azide (10/82 sensitive to 600 $\mu g$ $NaN_3$ $ml^{-1}$; 39/204 sensitive to 6,000 $\mu g$ $NaN_3$ $ml^{-1}$).

A previous investigation of cultures from continuous human tumour cell lines and a limited number of primary human tumour cultures demonstrated that the use of a metabolizable vital dye, INT, in conjunction with soft agar colony formation assays permitted discrimination between viable and non-viable groups of cells (Alley *et al.*, 1982). In the present evaluation INT was employed in the analysis of replicate "proliferation control" cultures derived from each of 135 consecutive evaluable human tumour specimens following 1, 5, and 10 or 1, 7, and 14 days of incubation. Elevated colony counts were observed in most cultures following 1 day of incubation: Counts ranged from 0 to 1031 INT-stained images with a median of 75; only 48 cultured specimens (35.5%) exhibited "acceptable" Day 1 colony counts and 44 specimens (33%) exhibited colony counts exceeding 100. Following 5 to 14 days' incubation only 22 specimens (16.3%) exhibited "significant" *in vitro* growth. Thus, tumour cell growth, if present, was overshadowed by a predominance of large cellular aggregates initially plated in the majority of viable specimens. Failure of the "conventional" colony formation assay to identify growth and azide-induced cytotoxicity prompted a major re-assessment of culture techniques.

*Assessment of tumour cell growth following use of a modified culture methodology*

It occurred to us that the presence of larger tumour cell aggregates and the apparent false-negative chemosensitivity profiles observed during performance of the conventional assay might in part be overcome by modification of the conventional culture methodology. In a subsequent assessment of 66 consecutive evaluable specimens using the modified procedure, 44 specimens (67%) exhibited acceptable Day 1 counts (median = 8) and only 8 specimens (12%) exhibited colony counts exceeding 100. During the course of 5 to 14 days' incubation 25 specimens (37.9%) exhibited significant increases in colony count ranging from 30 to 489 (median = 90, "plating" efficiencies ranging from 0.012 to 0.19%). Thus, coupled with the use of agarose, multiple-stage nylon mesh filtration resulted in a better appreciation of tumour cell growth within many primary cultures.

*Detection of chemosensitivity by the modified colony formation assay*

For a given specimen, drug sensitivity was assessed shortly following exhibition of significant growth in "proliferation control" cultures. In an initial assessment of 72 consecutive evaluable specimens using the modified colony formation assay 32 specimens (44%) exhibiting significant growth were successfully assayed. Sensitivity to sodium azide (600 $\mu g\ ml^{-1}$) was observed in 23 specimens by image analysis of INT-stained cultures, but in only 4 specimens by analysis of non-stained cultures. While sensitivity to one or more chemotherapeutic agents was observed in 19 specimens by image analysis of INT-stained cultures, image analysis of non-stained cultures (conventional) identified activity in only 8 specimens (Table I). Drug sensitivity (>70% inhibition of colony formation) was observed on 57 individual occasions utilizing the INT stain methodology (>90% inhibition was noted for 33), whereas analysis of non-stained cultures indicated drug sensitivity on only 25 individual occasions (>90% inhibition was noted for 2). When 12 additional specimen cultures were evaluated only by conventional image analysis due to inadequate numbers of INT-stained colonies, none of these cultures exhibited sensitivity to drugs or sodium azide. (The fact that several of the azide insensitive specimens were sensitive to one or more chemotherapeutic agents suggest that sodium azide may not be effectively cytotoxic in all primary human tumour cultures.)

In a subsequent assessment 123 consecutive evaluable specimens, 50 specimens (41%) exhibited significant *in vitro* growth (data not shown). Of these specimens, 36 exhibited sensitivity to one or more drugs by INT analysis, whereas only 7 of the specimens exhibited sensitivity by conventional image analysis. For all of the cultures evaluated by both methods to date with one exception (culture number 3229, Table I), INT analysis resulted in the detection of at least all agents identified by conventional image analysis.

Of the 195 (total) consecutive evaluable tumour specimens cultured using the new method, 82 (42%)

**Table I** Sensitivity of primary human tumour cell cultures to sodium azide and chemotherapeutic agents[a]

| | | *Conventional image analysis* | | *Image analysis following INT treatment* | |
|---|---|---|---|---|---|
| *Tumour source (culture number)* | *Number of drugs assayed* | *Sodium azide sensitivity* | *Number of "effective" drugs* | *Sodium azide sensitivity* | *Number of "effective" drugs* |
| Ovary (3166) | 13 | + | 4 | + | 5 |
| Lung (3167) | 10 | | 1 | + | 3 |
| Rectum (3173) | 9 | | | + | |
| Colon (3184) | 3 | | | | |
| Larynx (3185) | 11 | | | + | 2 |
| Endometrium (3186) | 10 | | | + | |
| Breast (3187) | 7 | | | | |
| Rectum (3190) | 1 | | | | 1 |
| Breast (3194) | 15 | | 1 | + | 4 |
| Ovary (3196B) | 1 | | | + | |
| Kidney (3199) | 20 | | | + | 3 |
| Colon (3209) | 20 | | | + | 1 |
| Breast (3215) | 3 | | | | |
| Ovary (3218) | 6 | | | + | |
| Melanoma (3219) | 17 | + | 3 | + | 3 |
| Breast (3221) | 4 | | | + | |
| Lung (3225) | 18 | | 2 | + | 6 |
| Ovary (3229) | 13 | | 5 | | 4 |
| Kidney (3231) | 1 | | | | |
| Colon (3234) | 3 | | | + | 2 |
| Breast (3237) | 3 | | | + | 1 |
| Neck (3240) | 11 | + | 3 | + | 7 |
| Ovary (3242) | 18 | | 7 | + | 7 |
| Colon (3246) | 7 | | | + | |
| Endometrium (3247) | 18 | | | + | 1 |
| Colon (3252) | 6 | | | | 3 |
| Endometrium (3253) | 7 | | | | 1 |
| Ovary (3255) | 19 | | | + | |
| Ovary (3258) | 8 | | | + | |
| Lung (3264) | 11 | | | + | 1 |
| Thyroid (3269) | 11 | | | | |
| Gastroesophagus (3270) | 10 | + | | + | 2 |

[a]Sodium azide sensitivity as well as drug "efficacy" refers to ≥70% inhibition of colony formation.

exhibited *in vitro* growth. While drug sensitivity was observed for 55 specimens (28%) utilizing INT stain, analysis of non-stained cultures indicated drug sensitivity for only 15 specimens (7.7%). Thus, the detection of growth and chemosensitivity of tumour cells cultured and analyzed according to the modified methodology was better than that observed using our previous methodology. Despite multi-step filtration of tumour cell suspensions in order to eliminate plating of large cellular aggregates, the majority of cultures still required the use of vital staining for the identification of active chemotherapeutic agents.

### *Microscopic profile of stained and non-stained cell groups in primary tumour cultures*

Microscopic inspection of cultures provided a useful means to examine the disparities between conventional image analysis and image analysis following INT treatment. Cultures exhibiting growth contained many densely-stained cell groups with diameters $>60\,\mu m$ (Figure 1a). By contrast, inspection of effectively drug-treated cultures often revealed proportionately fewer groups of cells and an overall lack of stain in cell groups less than as well as greater than $60\,\mu m$ in diameter (Figure 1b).

**Figure 1** Photomicrographs of a typical soft agarose cell culture following 9 days' incubation (INT stain). Many cell groups within control cultures (a) exceed 60 $\mu$m in diameter and exhibit densely-stained central regions as well as peripheral processes. By comparison, few cell groups within effectively drug-inhibited cultures (b) exceed 60 $\mu$m in diameter and very few are fully stained.

At low magnification such non-stained cell groups appeared to possess typical morphology of "colonies". Despite evidence of regional deterioration at higher magnification the overall morphology of a given cell group appeared "intact", perhaps due to physical stabilization provided by the surrounding culture matrix. That cell groups lacking INT stain are non-viable has been confirmed by application of other supravital stains (i.e., trypan blue and acridine orange/ethidium bromide) to the same culture and/or replicate cultures. The presence of drugs in culture at concentrations employed for routine screening does not appear to alter INT metabolism by viable tumour cell line cultures (Alley *et al.*, 1982).

Microscopic observations coupled with the fact that substantial increases in colony count and size occur between 1 and 5 days' incubation in drug-treated cultures suggests that doomed tumour cells can undergo significant growth before cytostasis and cytotoxicity imposed by drugs becomes manifest. Discrimination between viable and non-viable cell groups is particularly important in the assessment of primary tumour cell cultures because the number of viable cell groups is exceeded often greatly by the total non-viable cellularity and debris present in culture at the time of analysis. For this reason, use of INT (or perhaps other vital stains) appears to be mandatory for valid detection of growth as well as identification of effective chemotherapeutic agents.

*Alternate method of drug application in the chemosensitivity testing of primary tumour cell cultures*

Another aspect of conventional culture methodology which affects culture performance is the method for exposing cells to drugs. The usual protocol requires that prior to setting up bilayer cultures, aliquots of cell suspension be transferred to separate tubes, each containing a different drug, the same drug at different concentrations, and/or respective drug vehicles followed by the addition of several media supplements (Soehnlen *et al.*, 1980). After mixing the contents of each tube, aliquots of cell suspension are applied to replicate culture dishes using a different pipet for each tube. Each step not only imposes a degree of technical difficulty, but also reduces the accuracy of dispensing the same number of cells into each culture dish.

As an alternate approach for drug exposure, we assessed the potential utility of setting up all cultures from a single, large volume of cell suspension followed by the application of drug

**Figure 2** A comparison of drug activities in primary human tumour cell culture following two methods of drug application. The graph depicts paired mean % survival data gathered from nine individual specimen cultures (see text). N = sodium azide (600 $\mu g\,ml^{-1}$); F = 5-fluorouracil (10 $\mu g\,ml^{-1}$); M = mitomycin C (0.04 $\mu g\,ml^{-1}$); A = actinomycin D (0.01 $\mu g\,ml^{-1}$); D = doxorubicin (0.6 $\mu g\,ml^{-1}$); V = vinblastine (0.05 $\mu g\,ml^{-1}$); P = cisplatin (1.5 $\mu m\,ml^{-1}$).

directly to soft agarose culture surfaces. Preliminary experiments demonstrated that agarose matrix provides no significant barrier to the diffusion of any of 24 standard chemotherapeutic agents. Application of 100 $\mu l$ of concentrated drug solution (final culture concentration, $\mu g\,ml^{-1}$)* to continuous human tumour cell line cultures containing 1 ml base layer and 1 ml cellular layer resulted in $\geq$70% inhibition of colony formation ($\geq$90% inhibition in most cases) by all agents not requiring metabolic activation.

*actinomycin D (0.010), bisantrene (0.50), doxorubicin (0.60), L-alanosine (50), acridinyl anisidide (1.0), cytosine arabinoside (0.20), AZQ (1.0), carmustine (2.0), bleomycin (2.0), dibromodulcitol (5.0), galactitol (2.0), 5-fluorouracil (10), mitoguazone (50), chlorozotocin (2.0), hydroxyurea (60), methotrexate (1.0), mitomycin C (0.040), PALA (200), cisplatin (1.5), streptozotocin (6.0), triazinate (40), vinblastine (0.050), teniposide (10), etoposide (10).

In a subsequent experiment, drug activity following application by the incorporation technique was compared with that following application by the overlay technique for 7 agents in 9 primary human tumour cell cultures (2 colon, 4 kidney, and 3 ovary). Linear regression analysis of paired data is shown in Figure 2. All entries except 3 (circled) fall within 95% confidence limits of the line $Y = 0.914\ X + 22.1$, where $X$ represents the percent survival resulting from the drug overlay technique and $Y$ represents the percent survival measured by the drug incorporation technique ($r = 0.780$, $n = 42$, $P < 0.001$). A slope factor of 0.914 ($\pm$0.234, 95% CI) coupled with a $Y$ intercept of 22.1 ($\pm$13.0, 95% CI) suggests the drug overlay technique provides a somewhat more sensitive measure of drug effects overall.

The drug overlay technique was employed in the assessment of 145 consecutive evaluable human solid tumour specimens. Significant growth was observed in 73 specimen cultures, 50 of which were sensitive to sodium azide and 55 of which were sensitive to one or more chemotherapeutic agents. As shown in Table II, use of the drug overlay technique in this recent series of tumour cultures resulted in similar if not higher frequencies of detecting growth and chemosensitivity than were observed for the drug incorporation method in an earlier series of specimen cultures. While similar frequencies of azide and drug sensitivity were observed for the two methodologies when data is expressed relative to the number of assays (A), the fact that a higher incidence of growth occurred following drug overlay appears to have resulted in an overall higher frequency of chemosensitivity detection relative to the number of cultures (C). The diminished proliferation capacity of cells cultured by the drug incorporation technique may be due to the excessive mechanical manipulation of cells at elevated temperature required by that technique.

**Table II** Evaluability and chemosensitivity of primary human tumour cultures following two methods of drug application[a, b]

| *Criteria* | *Drug incorporation* | *Drug overlay* |
|---|---|---|
| Consecutive evaluable specimen cultures (C): | 195 | 145 |
| Cultures exhibiting significant growth and successfully assayed (A): | 82 (42.1% of C) | 76 (52.4% of C) |
| Azide sensitive: | 57 (69.5% of A; 29.2% of C) | 50 (65.8% of A; 34.5% of C) |
| Drug sensitive: | 55 (67% of A; 28.2% of C) | 55 (72.4% of A; 37.9% of C) |
| Azide and/or drug sensitive: | 71 (86.6% of A; 36.4% of C) | 63 (82.9% of A; 43.4% of C) |

[a]Data from two separate phases of study are summarized (see text). Table entries indicate the number and normalized frequencies of specimen cultures meeting each criterion.

[b]Sensitivity refers to ≥70% inhibition of colony formation by sodium azide (600 $\mu$g ml$^{-1}$) or chemotherapeutic agents present at clinically achievable concentrations.

The observation that sodium azide was not highly cytotoxic in ~30% of the specimen cultures assayed prompted us to investigate the possible utility of other cytotoxic control agents. Of 50 consecutively assayed specimen cultures, all were sensitive (>95% inhibition of "colony" formation in all but 2 specimen cultures) to mercuric chloride (100 $\mu$g ml$^{-1}$, final culture concentration), whereas only 34 (68%) were azide-sensitive (>70% inhibition). These data indicate that mercuric chloride is a highly effective "cytotoxicity control" agent.

### Discussion

Five years following the first publication concerning drug sensitivity testing in primary human tumour cell cultures by Salmon and colleagues (1978), critical assessments of the "human tumour stem cell assay" have begun to appear in the literature. Some have presented "theoretical" considerations of "stem cell" biology, whereas others have discussed the relevance of *in vitro* drug sensitivity to the clinical management of patients (e.g., Selby *et al.*, 1983; Editorial, 1983). Our perspective concerning the "human tumour stem cell assay" has been more pragmatic and constructively critical: that of a laboratory investigating whether it is possible to correct numerous technical problems with its conventional performance. The present manuscript describes method modifications and specific "control" measures which we feel are essential for valid detection of growth and drug sensitivity determinations of cultured human solid tumour cells.

A key observation at a practical level (as well as possibly theoretical "stem cell" level) is the nearly universal presence of aggregated cells in culture suspensions prepared by either enzymatic or mechanical disaggregation techniques. As a consequence, many "colonies" observed following one to two weeks' culture incubation arise from the enlargement of pre-existing cell aggregates (Agrez *et al.*, 1982*b*). Because of the initial presence of such cell aggregates in soft agar matrix, a valid assay must "account" for them. First, a major effort must be made to eliminate large cellular aggregates (>60 $\mu$m diameter) from tumour cell suspensions. In the present study, a series of filtrations accomplished this goal without imposing significant reductions in tumour cell yield. Second, appropriate "proliferation control" cultures must be evaluated the day after the cultures are initiated in order to determine the size and number of cellular aggregates which are invariably present. Computerized image analysis of INT-stained cultures permits objective achievement of this task. (It is important to note that cell groups in non-stained cultures often exhibit minimal optical density during the early stages of culture and, therefore, are inefficiently detected by computerized image analysis.) Third, soft agar colony formation assays utilized for drug screening of necessity must include one or more "cytotoxicity control" compounds (e.g., sodium azide or mercuric chloride). "Cytotoxicity control" cultures in conjunction with vital staining must demonstrate >90% inhibition of colony formation before a given assay can be considered valid for drug screening. Finally, since cellular aggregates which undergo spontaneous or drug-induced death persist in soft agar culture, we believe that use of INT which provides a means to distinguish viable groups of cells from non-viable cell groups and debris from detection (Alley *et al.*, 1982) markedly improves optical determinations of growth and drug sensitivity in this assay.

Previous studies utilizing the "human tumour stem cell assay" have employed either "one hour" exposure of tumour cells to drugs prior to culture or "continuous" exposure to drugs incorporated within the soft agar culture matrix (Soehnlen *et al.*, 1980). The alternate method of drug exposure assessed in the present study, culture surface application, appears to be far less cumbersome. Also, such a technique eliminates the possibility that a "mechanical" feature of the drug application itself could contribute to the failure of cell growth.

None of the published reports which suggest a possible role for the "human tumour stem cell assay" in clinical selection of anticancer therapy has acknowledged the series of technical problems described herein. We, as others, believe these reports should be viewed with an attitude of "cautious skepticism" (Rupniak & Hill, 1980; Bertoncello *et al.*, 1980; Lieber & Kovach, 1982) Editorial, 1982. The incorporation of aggregated cells into culture as well as the persistence of non-viable cell groups and debris in soft agar matrix undoubtedly contribute to false-negative *in vitro* drug sensitivity profiles. Indeed, most previously reported *in vitro* experiments utilizing the conventional "human tumour stem cell assay" to test drugs failed to identify significant *in vitro* drug sensitivity. Since commonly tested human tumours (colon, breast, renal, melanoma, etc.) are clinically resistant to most single agent therapies and since the majority of contributions to "positive" correlation have been between *in vitro* drug resistance and clinical resistance, it is not surprising to us that excellent *in vitro/in vivo* correlations have been reported thus far.

Whether the assessment of short-term *in vitro* growth by tumour cells exposed to anticancer drugs will prove to be clinically useful remains to be determined by prospective laboratory and laboratory/clinic correlative studies. At present it is clear that multiple technical problems are associated with conventional performance of the "human tumour stem cell assay". Nevertheless, specific modifications of this assay coupled with appropriate "control" measures appear to improve detection of *in vitro* tumour cell growth and drug sensitivity.

The authors thank Mary Adams, Linda Foster, Barbara Furlow, Sue Gossman, Sharon Guy, Dane Mathieson, Donna Sculley, Cindy Uhl and Carol White for sustained efforts in the culture and analysis of solid tumour specimens and Shelly Nicklay for preparation of the manuscript.

### References

AGREZ, M.V., KOVACH, J.S., BEART, R.W. Jr., RUBIN, J., MOERTEL, C.G. & LIEBER, M.M. (1982*a*). Human colorectal carcinoma: Patterns of sensitivity to chemotherapeutic agents in the human tumor stem cell assay. *J. Surg. Oncol.*, **20**, 187.

AGREZ, M.V., KOVACH, J.S. & LIEBER, M.M. (1982*b*) Cell aggregates in the soft agar "human tumour stem-cell assay". *Br. J. Cancer*, **46**, 880.

ALLEY, M.C., UHL, C.B. & LIEBER, M.M. (1982). Improved detection of drug cytotoxicity in the soft-agar colony formation assay through use of a metabolizable tetrazolium salt. *Life Sci.*, **31**, 3071.

BERTONCELLO, I., BRADLEY, T.R., CAMPBELL, J.J. & 6 others. (1982). Limitations of the clonal agar assay for the assessment of primary human ovarian tumor biopsies. *Br. J. Cancer*, **45**, 803.

BOL, S., VAN DEN ENGH, G. & VISSER, J. (1977). A technique for staining haemopoietic colonies in agar cultures. *Exp. Hematol.*, **5**, 551.

EDITORIAL. (1982). Clonogenic assays for the chemotherapeutic sensitivity of human tumors. *Lancet*, **i**, 780.

EDITORIAL. (1983). Human tumor stem-cell assay. *N. Engl. J. Med.*, **308**, 1478.

GARVEY, J.S., CREMER, N.E. & SUSSDORF, D.H. (Eds.) (1979). *Methods in Immunology*, 3rd Edn, Reading, Massachusetts: W.A. Benjamin, Inc.

HAMBURGER, A.W. & SALMON, S.E. (1977*a*). Primary bioassay of human tumor stem cells. *Science*, **197**, 461.

HAMBURGER, A.W. & SALMON, S.E. (1977*b*). Primary bioassay of human myeloma stem cells. *J. Clin. Invest.*, **30**, 846.

JERNE, N.K., HENRY, C., NORDIN, A.A., FUJI, H., KOROS, A.M.C. & LEFKOVITS, I. (1974). Plaque forming cells; methodology and theory. *Transplant. Rev.*, **18**, 130.

LIEBER, M.M. & KOVACH, J.S. (1982). Soft agar colony formation assay for chemotherapy sensitivity testing of human solid tumours. *Mayo Clinic Proc.*, **57**, 527.

PAVELIC, Z.P., SLOCUM, H.K., RUSTUM, Y.M. & 5 others. (1980). Growth of cell colonies in soft agar from biopsies of different human solid tumors. *Cancer Res.*, **40**, 4051.

RUPNIAK, H.T. & HILL, B.T. (1980). The poor cloning ability in agar of human tumor cells from biopsies of primary tumors. *Cell Biol. Int. Reps.*, **4**, 479.

SALMON, S.E., HAMBURGER, A.W., SOEHNLEN, B., DURIE, B.G.M., ALBERTS, D.S. & MOON, T.E. (1978). Quantitation of differential sensitivity of human tumor stem cells to anticancer drugs. *N. Engl. J. Med.*, **298**, 1321.

SALMON, S.E. (ed.) (1980). Cloning of human tumor stem cells. *Prog. Clin. Biol. Res.*, **48**, 1.

SCHAEFFER, W.I. & FRIEND, K. (1976). Efficient detection of soft-agar grown colonies using a tetrazolium salt. *Cancer Lett.*, **1**, 259.

SELBY, P.J., BUICK, R.N. & TANNOCK, I. (1983). A critical appraisal of the "human tumor stem-cell assay". *N. Engl. J. Med.*, **308**, 129.

SOEHNLEN, B., YOUNG, L. & LIU, R. (1980). Standard laboratory procedures for *in vitro* assay of human tumor stem cells. *Prog. Clin. Biol. Res.*, **48**, 331.